# Development of the DNA-based voltammetric biosensor for detection of vincristine as anticancer drug

**DOI:** 10.3389/fchem.2022.1060706

**Published:** 2023-01-09

**Authors:** Mahmoud Abbasi, Fahad Alsaikhan, Rasha Fadhel Obaid, Shohreh Jahani, Saeed Biroudian, Maziar Oveisee, Mohammad Reza Arab, Zahra Aramesh-Boroujeni, Mohammad Mehdi Foroughi

**Affiliations:** ^1^ Medical Ethics and Law Research Center, Shahid Beheshti University of Medical Sciences, Tehran, Iran; ^2^ College of Pharmacy, Prince Sattam Bin Abdulaziz University, Alkharj, Saudi Arabia; ^3^ Department of Biomedical Engineering, Al-Mustaqbal University College, Babylon, Iraq; ^4^ Noncommunicable Diseases Research Center, Bam University of Medical Sciences, Bam, Iran; ^5^ Department of Medical Ethics, Medical School, Iran University of Medical Sciences, Tehran, Iran; ^6^ Orthopedic Department, Bam University of Medical Sciences, Bam, Iran; ^7^ Department of Medical, Bam University of Medical Sciences, Bam, Iran; ^8^ Department of Chemistry, University of Isfahan, Isfahan, Iran; ^9^ Department of Chemistry, Kerman Branch, Islamic Azad University, Kerman, Iran

**Keywords:** vincristine, polypyrrole, peony-like CuO:Tb 3+ nanostructure, DNA biosensor, voltammetry

## Abstract

In the article presented herein, a deoxyribonucleic acid (DNA) biosensor is introduced for Vincristine determination in pharmaceutical preparations based on the modification of screen printed electrode (SPE) with double-stranded DNA (ds-DNA), polypyrrole (PP), peony-like CuO:Tb^3+^ nanostructure (P-L CuO:Tb^3+^ NS). The developed sensor indicated a wide linear response to Vincristine concentration ranged from 1.0 nM to 400.0 μM with a limit of detection as low as .21 nM. The intercalation of Vincristine with DNA guanine led to the response. The optimized parameters for the biosensor performance were ds-DNA/Vincristine interaction time, DNA concentration and type of buffer solution. The docking investigation confirm the minor groove interaction between guanine base at surface of or ds-DNA/PP/P-L CuO:Tb^3+^ NS/SPE and Vincristine. The proposed sensor could successfully determine Vincristine in Vincristine injections and biological fluids, with acceptable obtains.

## 1 Introduction

**FIGURE 1:**
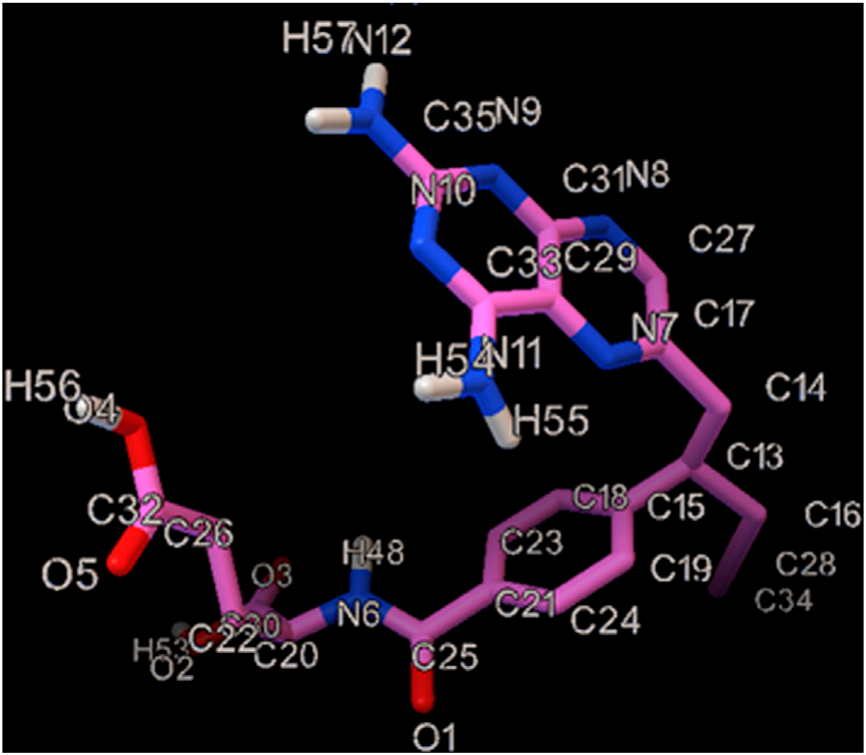
The illustration of the chemical structure of Vincristine with the numbered atoms.

Vincristine (Figure 1) is placed in a class of drugs called the vinca alkaloids, which is extracted from *Catharanthus roseus*, and used as a chemotherapy drug (Khan et al., 2022). Vincristine is applied for treating a various type of cancers such as acute lymphocytic leukemia, acute myeloid leukemia, neuroblastoma, Hodgkin’s disease, rhabdomyosarcoma, Wilms’ tumor and small cell lung cancer (Li et al., 2021; Filippi-Chiela et al., 2022). Some VCR-caused complications are headaches, hair loss, difficulty in walking, constipation, change in sensation, neuropathic pain, lung damage and lowered white blood cells (Childress et al., 2022; Mujib et al., 2022). Therefore, it is essential to quantify the VCR in the biological specimens like urine and plasma. There are diverse analytical methods in this regard, including liquid chromatography together with electrochemical or ultraviolet determination, liquid chromatography-mass spectrometry and LC–MS/MS (Gupta et al., 2005; Amila Jeewantha et al., 2017; Kiani et al., 2018; Jin et al., 2021).

Among these, the electrochemical biosensors have some merits like selectivity, sensitivity, cost-effectiveness, rapidity and simplicity (Foroughi and Jahani, 2021; Zhang et al., 2022). The screen printed electrode (SPE) can be used to produce a disposable equipment (Núñez et al., 2021). Deoxyribonucleic acid (DNA) biochemical biosensors concentrate molecules with affinity for nucleic acids to assess the electronic surface using the DNA layer selectivity. Alterations in the redox attributes of DNA (in the guanine oxidation) are examined to study the interactions between DNA and analytes in these biosensors (Moarefdoust et al., 2022a; Cao et al., 2022; Fang et al., 2022).

The electrode surface modification can boost the function of the electrode to establish a suitable substrate for the stabilization of biomolecules and to decrease the charge transfer resistance on the sensor surface (Li et al., 2016; Zhou et al., 2018; Farvardin et al., 2020; Iftikhar et al., 2021a; Gao et al., 2022; Jahani et al., 2022; Jia et al., 2022). The sensors can be produced by a majority of metals, metal oxides and alloys (Fathi et al., 2020; Vakili Fathabadi et al., 2020; Ejaz et al., 2021; Anqi et al., 2022; Chen et al., 2022; Duan et al., 2023; Iftikhar et al., 2022). Several materials are noble metals or toxic metals, others with slow kinetics and negligible selectivity (Zhang et al., 2016a; Yang et al., 2017; Iftikhar et al., 2021b; Foroughi et al., 2021; Moarefdoust et al., 2022b; Kumar et al., 2022). Copper oxide (CuO) is a p-type semiconductor, has a 1.2-eV bandgap and can be utilized in the structure of batteries, catalysis, biosensors and gas sensors (Park et al., 2014; Yang et al., 2014; Hu and Liu, 2015). The merits of CuO for sensor applications can be attributed to non-toxicity, cost-effectiveness, easy fabrication, specific capacitances and facile storage (Yang et al., 2012). The electrocatalytic traits of CuO can be reinforced through the combination of CuO with highly conductive materials like rare Earth metals, gold (Au), silver (Ag), carbon nanotubes (CNTs) and graphene to generate composite materials (Dung et al., 2013; Zheng et al., 2014; Dong et al., 2015; Tian et al., 2015).

The current attempt was made to fabricate a selective and sensitive method to determine the Vincristine. The literature review revealed that there is no study so far evaluating determination of Vincristine based on electrochemical DNA. The sensor was modified with double-stranded DNA (ds-DNA), polypyrrole (PP) and peony-like CuO:Tb^3+^ nanostructure (P-L CuO:Tb^3+^ NS) to determine nano-molar Vincristine. The practical potential of the proposed ds-DNA/PP/P-L CuO:Tb^3+^ NS/SPE sensor was verified by determining Vincristine in urine, blood serum and injection. We found outstanding advantages for our biosensor, including an impressive sensitivity, cost-effective, admirable reproducibility fast response, and narrow limit of detection in spite of the presence of various interferants. In addition, the findings of this study are significant because ds-DNA/PP/P-L CuO:Tb^3+^ NS/SPE provided appreciable analytical behavior and sensitivity when comparing with counterpart electrochemical and non-electrochemical methods previously introduced for Vincristine determining.

## 2 Experimental

### 2.1 Chemicals and devices

Vincristine (>99.0%), sodium nitrate (NaNO_3_, = 99.0%), copper nitrate trihydrate (Cu(NO_3_)_2_.3H_2_O, >99.0%), absolute ethanol (=99.8%), NaOH (>97%), 28% ammonia and terbium chloride hexahydrate (TbCl_3_.6H_2_O, >99.0%) belonged to Sigma–Aldrich Company (Germany). All solutions were freshly prepared by double distilled water (DDW). To obtain 1.0 mM Vincristine stock solution, Vincristine (824.96 mg) was dissolved in water solution. The freshly prepared human blood serum and urine samples were from Pasteur Bam Hospital (Bam, Iran). The 1-mg/ml Vincristine ampoule was from Nanodaru Pajuhan Pardis Co. (Tehran; Iran). All measurements of electrochemical impedance spectroscopy (EIS), voltammetry were performed by a SAMA 500 Electro-analyzer (Isfahan; Iran). The three-electrode cell system contained one working electrode (SPE), one axillary electrode (platinum wire) and one reference electrode (SCE). All pH values were measured by a digital ELICO LI 120 pH meter. All X-ray powder diffraction (XRD) findings belonged to a Philips PC-APD X-ray diffractometer (the Netherlands). EM 3200 SEM and KYKY Scanning Electron Microscopy-Energy Dispersive Spectroscopy (SEM-EDS; China) characterized the modifier.

### 2.2 Production of peony-like CuO:Tb^3+^ nanostructure (P-L CuO:Tb^3+^ NS)

A simple hydrothermal protocol was applied to construct the P-L CuO:Tb^3+^ NS. Thus, 1.2 g of copper nitrate trihydrate and .1 g of terbium chloride hexahydrate were poured in 100 ml of ethanol. Then, the solution was added with 25 ml of 28% ammonia and 20 ml of 1.0 M NaOH as dropwise, followed by appending 10 g of NaNO_3_. Next, the solution was autoclaved in a 250-ml Teflon-lined stainless steel device. After that, the mix was heated in an electric oven at 140°C for 24 h, the result of which was a black product that was gathered by washing with water, performing centrifugation and drying at 80°C.

### 2.3 Modification of electrode surface

The bare SPE (BSPE) was exposed to Piranha solution (H_2_SO_4_:H_2_O_2_, 3:1 as v/v) and ultra-sonicated for 15 s. Next, 1 mg of P-L CuO:Tb^3+^ NS was dispersed to 1 ml of DDW and ultra-sonicated for 1 hour, followed by adding .1 M pyrrole to the obtained solution. The pyrrole electro-polymerization on the SPE surface was carried out using the cyclic voltammetry (CV) (30 potential cycles between .0 and .8 V at 100 mV/s scan rate) (Foroughi and Jahani, 2021). To prepare the dsDNA solution, 90.0 mg of fish sperm DNA was dissolved in 1 L of DDW in ultrasonic bath to obtain a homogeneous solution. Afterwards, a certain amount of produced solution (5 µL) was casted on the PP/P-L CuO:Tb^3+^ NS/SPE (Moarefdoust et al., 2022b).

### 2.4 Sample treatment and detection

Vincristine was electroanalytically detected in the real pharmaceutical formulations and human blood serum and urine samples. 1 ml of Vincristine Sulfate (1 mg/ml solution) for injection diluted to 100 ml in acetate buffer solution at the pH value of 4.8. The standard solutions of Vincristine was spiked in the pharmaceutical formulations to perform the recovery tests of their determination. No pretreatment step was conducted for the detection of Vincristine in the refrigerated human blood serum and urine samples collected from a Pasteur Bam Hospital (Bam, Iran).

### 2.5 Molecular docking study

A molecular docking investigation was conducted as part of a biological assay to predict the mode of binding of vincristine anticancer drug inside the DNA receptor. From the Brook haven protein data bank, the crystal structures of DNA duplex (entry codes 1BNA and with sequence d (CGCGAATTCGCG)_2_ dodecamer) was downloaded.

## 3 Result and discussion

### 3.1 Characterization of P-L CuO:Tb^3+^ NS

**FIGURE 2:**
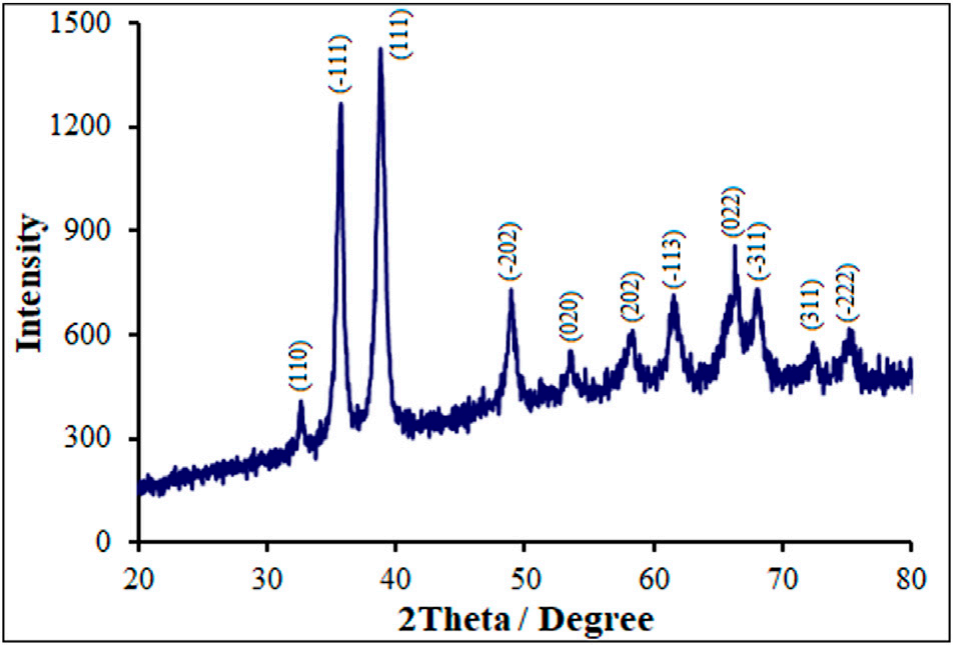
XRD pattern of P-L CuO:Tb^3+^ NS.

Figure 2 illustrates the XRD spectra obtained from pure and Tb-doped CuO nanostructures. The XRD spectra for all specimens are similar to those for monoclinic CuO nanostructures, JCPDS:895,898 (Uma Maheswari et al., 2018). The diffraction peaks at 2θ for monoclinic CuO include (−222), (311), (−311), (022), (−113), (202), (020), (−202), (111), (−111) and (110) corresponding to 75.36°,72.68°, 68.12°, 66.24°, 61.52°, 58.44°, 53.68°, 48.92°, 38.76°, 35.72° and 32.56°, sequentially. Higher degree switch is there for P-L CuO:Tb^3+^ NS at peaks (−202), (022) and (−311), probably because of Tb incorporation in CuO that imposes internal stress owing to its larger atomic radii (175 p.m.) when comparing with that for Cu (128 p.m.). It rendered also for the formation of defects. Replacing dopants of a lower atomic radius in the host lattice results in a greater angle peak shift. In our study, paradoxically, the peak angle shift is higher and the end of distance d is less observed for dopants that their atomic radius is larger than that of host copper, which may be related to further internal tensile stress caused by dopants and defects in the network, probably resulting in an abnormal shift in the greater angle peak and lower end of distance d found in XRD (Jansi Rani et al., 2020). The XRD had no secondary peaks related to dopant, confirming the fabrication of single-phase CuO NSs. According to the XRD patterns, the appeared diffraction peaks are related only to CuO formation. There were no other peaks related to Tb oxides. The lower shift of angle peaks related to greater atomic radii of dopant ion has been evaluated in detail.

**FIGURE 3:**
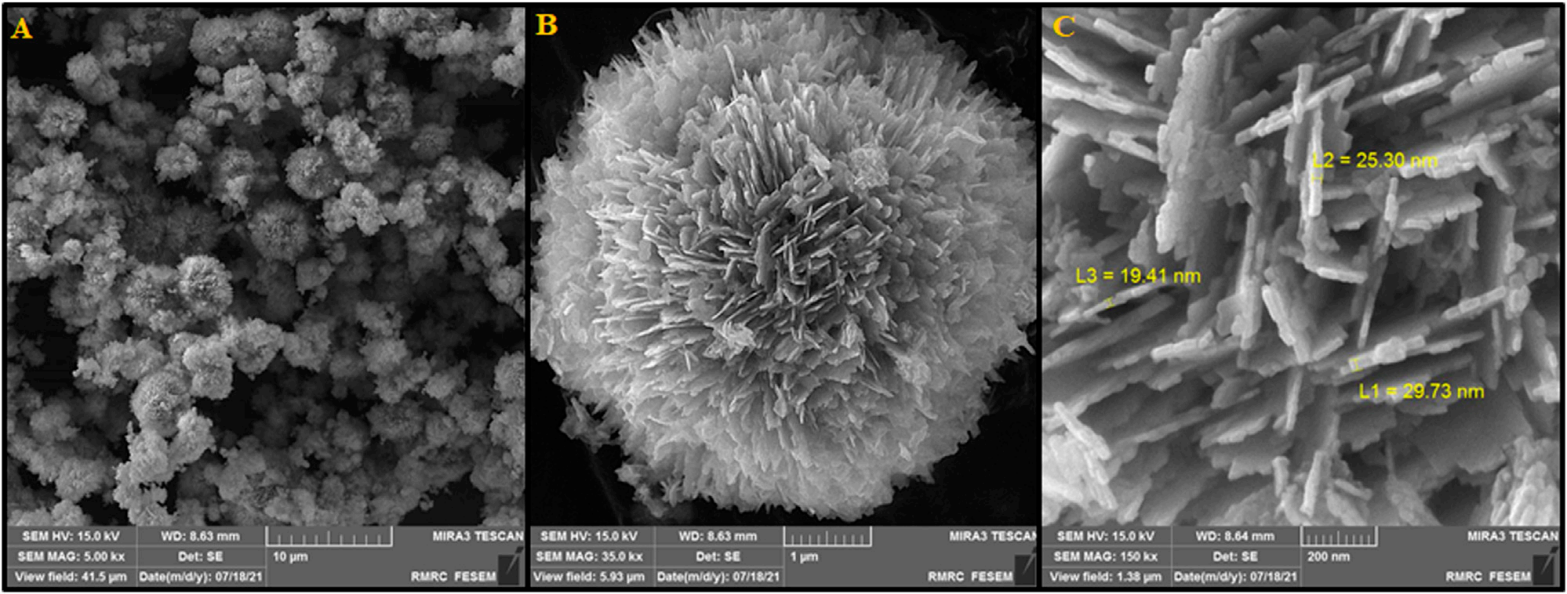
**(A)** FESEM image, **(B)** and **(C)** High resolution FESEM image of P-L CuO:Tb^3+^ NS.

Figure 3 depicts SEM images exhibiting the morphology and size of fabricated P-L CuO:Tb^3+^ NS. As shown in Figure 3A, a uniform peony-like morphology can be observed for these 5-μm fabricated nanoflowers regularly stacked by CuO:Tb^3+^ sheets. Figures 3B, C, with greater magnification, shows clearer shape of CuO:Tb^3+^ NS. Images show a unique hierarchical flower-like architecture containing ultra-thin nano-sheets with a mean thickness of 20 nm. The images show the uniform distribution of the nano-sheets along the radius throughout the sample, which resulted in the formation of a flower-like structure with abundant spaces between the petals. This structure, due to its larger specific surface area, can promise the construction of catalysts with unique applications.

**FIGURE 4:**
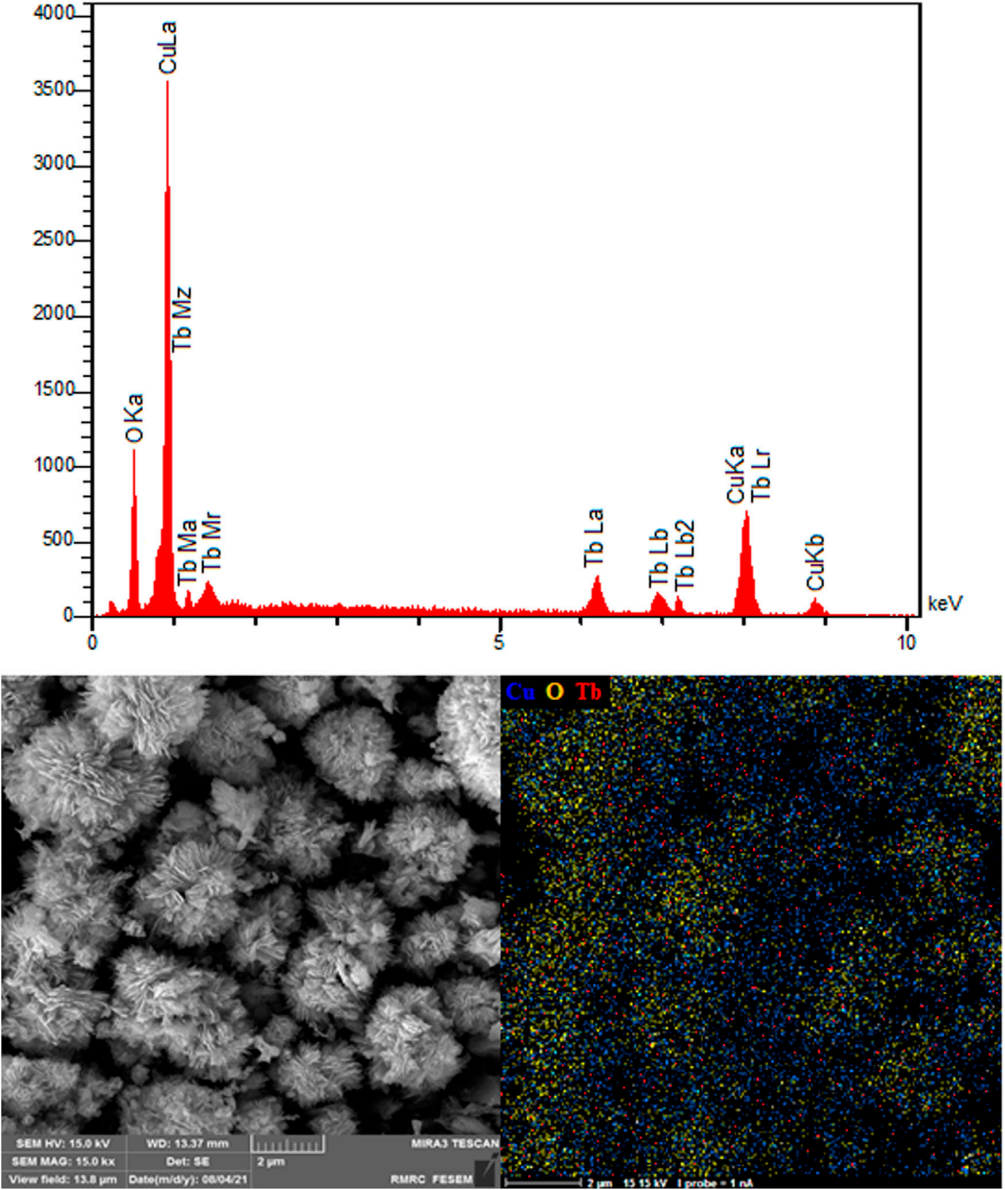
EDS spectra and elemental mapping of P-L CuO:Tb^3+^ NS.

Figure 4 verifies the elements of as-synthesized NSs and exhibits the electrocatalyst purities. The existence of Cu, Tb and O is evident. The EDS patterns display the production of NSs without any impurity and with high quality, probably supporting the statement of sample purity in the XRD spectrum. The EDS mapping analysis was also applied to determine the spatial dispersion of Cu, Tb and O. Figure 4 illustrates entire dispersion of Tb (red zone), Cu (blue zone) and O (yellow zone) throughout the area, indicating evident reasons for uniform dispersion in the P-L CuO:Tb^3+^ NS.

### 3.2 Electrochemical behaviors of modified electrode

**FIGURE 5:**
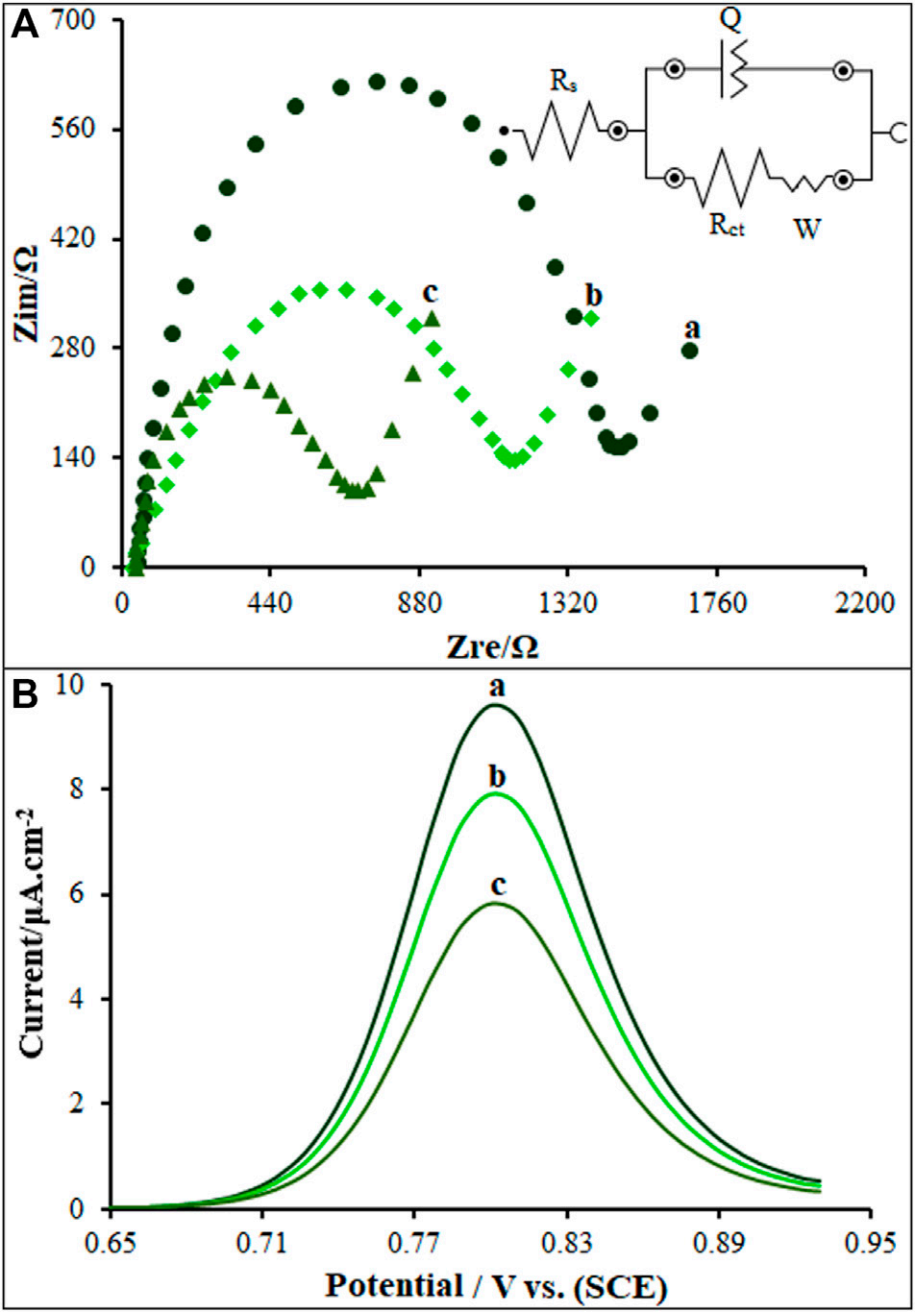
**(A)** Nyquist diagrams and the equivalent circuit of unmodified SPE **(A)**, PP/P-L CuO NS/SPE **(B)** and PP/P-L CuO:Tb^3+^ NS/SPE **(C)** in 1.0 mM [Fe(CN)_6_]^3-/4-^ (.1 M KCl) **(B)** DP voltammograms of ds-DNA/PP/P-L CuO:Tb^3+^ NS/SPE in absence **(A)** and presence of 35.0 µM **(B)** and 60.0 µM **(C)** Vincristine.

The modified electrode was assessed for the electrochemical behaviors by the EIS (Figure 5A) in 1.0 mmol/L [Fe(CN)_6_]^3-/4-^/0.1 M KCl electrolyte, in comparison to unmodified SPE, PP/P-L CuO NS/SPE and PP/P-L CuO:Tb^3+^ NS/SPE. The charge-transfer resistance (Rct) was estimated at approximately 1,482 Ω for the SPE according to Nyquist diagram. In the identical experimental circumstances, there was a reduction in the semicircle diameter on the PP/P-L CuO:Tb^3+^ NS/SPE and PP/P-L CuO NS/SPE, with the Rct values of approximately 660 and 1,143 Ω. Anchoring PP/P-L CuO:Tb^3+^ NS on the SPE surface caused an increase in electrical conductivity of electrode surface and a decrease in mass-transfer resistance during the redox process.

### 3.3 Intercalation of ds-DNA and vincristine

The differential pulse voltammetry’s (DPVs) were recorded for as-fabricated sensor under inclusion and exclusion of the Vincristine for the exploration of its intercalation with ds-DNA on the ds-DNA/PP/P-L CuO:Tb^3+^ NS/SPE surface (Figure 5B). As seen in Figure 5B (curve a), the Vincristine exclusion created an oxidation signal at 805 mV with an oxidation current of 9.6 µA in .5 M acetate buffer solution (pH 4.8). In the inclusion of 35.0 and 60.0 µM of Vincristine (in Tris-HCl buffer; pH 7.4), the ds-DNA/PP/P-L CuO:Tb^3+^ NS/SPE was placed in the solution and then stirred for 12 min. Next, .5 M acetate buffer solution (pH 4.8) was replaced by Tris-HCl, followed by recording the DPV for ds-DNA/PP/P-L CuO:Tb^3+^ NS/SPE. According to data in Figure 5B (curves b and c), the 35.0 and 60.0 µM Vincristine solutions had the oxidation currents of 7.9 and 5.8 µA, respectively. Vincristine declined the oxidation signal of ds-DNA on the ds-DNA/PP/P-L CuO:Tb^3+^ NS/SPE surface. Therefore, the intercalation of Vincristine with ds-DNA was validated on as-fabricated sensor surface.

### 3.4 Optimization of determinants

**FIGURE 6:**
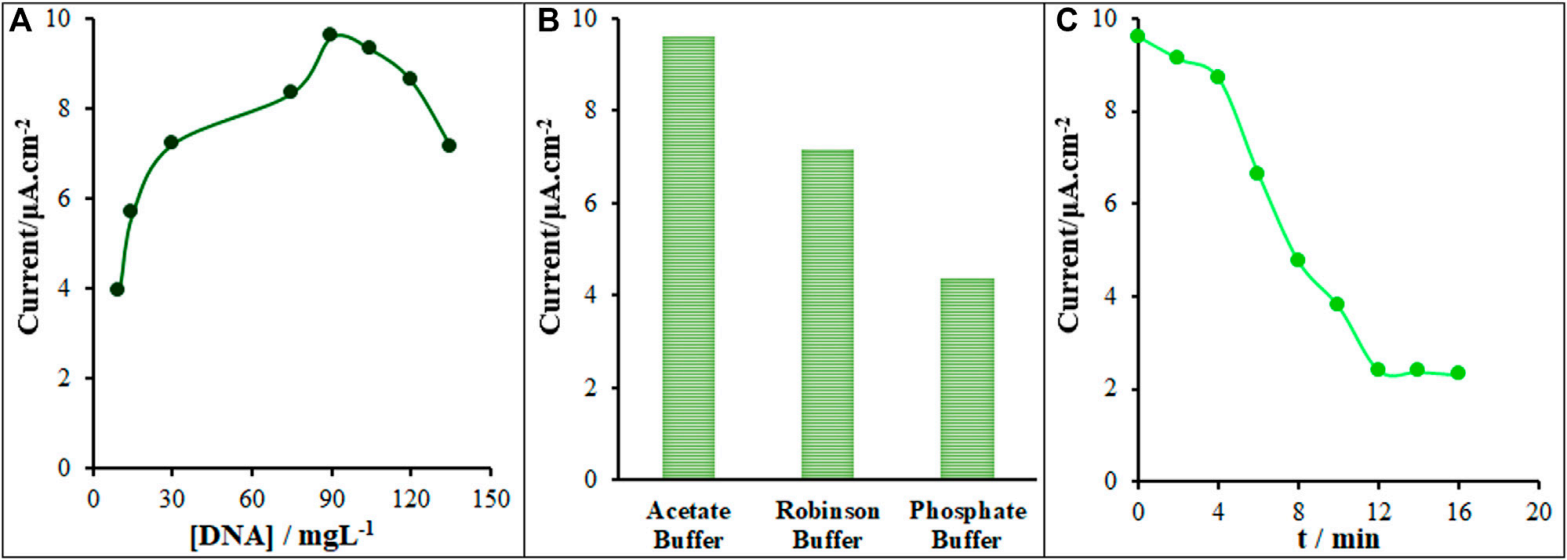
**(A)** Current *versus* ds-DNA concentration (10.0, 15.0, 30.0, 75.0, 90.0, 105.0, 120.0 and 135.0 mg/L) plot **(B)** Current recorded on ds-DNA/PP/P-L CuO:Tb^3+^ NS/SPE vs type of buffer at optimum condition (90.0 mg/L ds-DNA) **(C)** Effect of the time duration of 350.0 µM Vincristine on the guanine oxidation signal recorded on ds-DNA/PP/P-L CuO:Tb^3+^ NS/SPE (*n* = 5).

The determining parameters in the optimization of biosensor activity were ds-DNA concentration, intercalation time and type of buffer solution. Variable ds-DNA contents were applied to construct the ds-DNA/PP/P-L CuO:Tb^3+^ NS/SPE according to Section 2.3. As seen in Figure 6A, there was an increase in the guanine oxidation current with increasing ds-DNA content on the ds-DNA/PP/P-L CuO:Tb^3+^ NS/SPE surface. The highest oxidation current was related to starting ds-DNA concentration of 90.0 mg/L, as shown in Figure 6A. At higher concentrations, the oxidation signal of guanine was stable compared to ds-DNA, which means the PP/P-L CuO:Tb^3+^ NS/SPE surface was saturated by being occupied with ds-DNA molecules. Hence, the optimized starting concentration was selected to be 90.0 mg/L in the fabrication of ds-DNA/PP/P-L CuO:Tb^3+^ NS/SPE. Then, the signal of ds-DNA guanine was recorded at different buffer solutions of Britton–Robinson, acetate and phosphate buffer solutions with the pH 4.8. Figure 6B shows the oxidation currents of 7.15, 9.60 and 4.36 µA recorded for ds-DNA/PP/P-L CuO:Tb^3+^ NS/SPE in Britton–Robinson acetate, and phosphate buffer solutions, successively. As seen, the maximum sensitivity was found in the acetate buffer, thereby it was the solution selected for next testing. The last optimization step was related to the incubation time of the sensor. Thus, the modified electrode was exposed to the Vincristine solution while stirring for variable times. Finally, the interaction of ds-DNA with Vincristine on the ds-DNA/PP/P-L CuO:Tb^3+^ NS/SPE surface was completed during 12 min, and so this time was selected to be optimal for next testing (Figure 6C).

### 3.5 Analytical experiments

**FIGURE 7:**
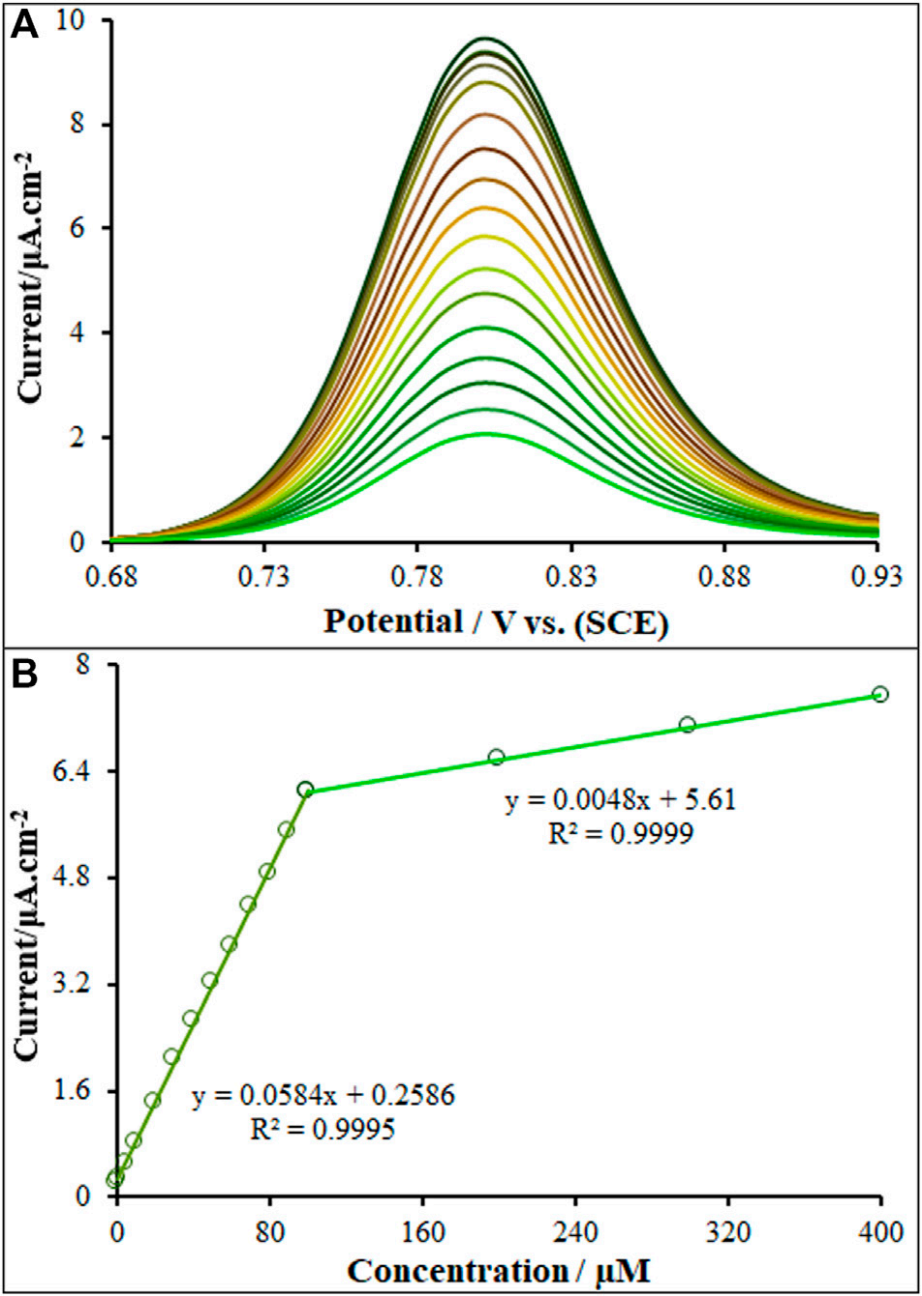
**(A)** DP voltammograms of ds-DNA/PP/P-L CuO:Tb^3+^ NS/SPE in the presence of increasing concentrations (.0, .001; 1.0; 5.0; 10.0; 20.0; 30.0; 40.0; 50.0; 60.0; 70.0; 80.0; 90.0; 100.0; 200.0, 300.0 and 400.0; µM) of Vincristine **(B)** ΔI *vs.* Vincristine concentration (*n* = 5) plot.

A general, pulse techniques, such as DPV, are more sensitive than the linear sweep methods because there is minimization of the capacitive current. In turn, CV is most commonly used for exploratory purposes. In DPV, small amplitude, short pulses are superimposed on a linear ramp. Current is measured before the application of the pulse and at the end of each pulse, and the difference between the currents is calculated. This procedure effectively reduces the background current due to the direct current (DC) ramp, and thus this procedure results in a Faradaic current free of most capacitive current. The major advantage of DPV is low capacitive current, which leads to high sensitivity. In inclusion and exclusion of Vincristine at variable concentrations, the DPVs were obtained for ds-DNA/PP/P-L CuO:Tb^3+^ NS/SPE (Figure 7A). In order to achieve the higher analytical response (anodic current), the optimal conditions for DPV measurements were as follow: ABS, pH 4.8, modulation amplitude of .02505 V, modulation time of 30 ms, interval time of 200 ms, step potential of 10 mV, initial potential = 680 mV and end potential of 930 mV. The net oxidation current of guanine in relation to ds-DNA on the ds-DNA/PP/P-L CuO:Tb^3+^ NS/SPE surface (net current refers to a difference between oxidation current in exposure or non-exposure to drug) had two linear regression equation is demonstrated by y = .0584x + .2586 from .001 µM to 100.0 µM linear range with *R* ^2^ = .9995 while the other linear range (100.0–400.0 µM) represents linear regression equation of y = .0048x + 5.61 (*R* ^2^ = .999) for Vincristine concentration. The limit of detections (LOD) as low as .21 nM and 2.39 nM (LOD = 3S_b_/m; where, S_b_ refers to the standard deviation of blank (*n* = 5) and m means the slope of linear dynamic range (LDR)), as seen in Figure 7B. According to the result, the high slope of the curve at low concentrations indicates the fact that the electrode provides enough active sites for vincristine. Whereas, the low slope value of the curve at high concentrations highlights relatively limited active sites for concentrated vincristine.

### 3.6 Comparison of proposed biosensor with other reported analytical methods for the determination of vincristine

The comparison of analytical efficacy between as-fabricated electrode and other non-electrochemical and electrochemical methods was performed for Vincristine (Table 1) (Yong et al., 2004; Gupta et al., 2005; Babkina and Ulakhovich, 2011; Zhang et al., 2016b; Amila Jeewantha et al., 2017; Kiani et al., 2018; Saify Nabiabad and Amini, 2020; Jin et al., 2021). Based on Table 1, the detection limit and linear range of as-fabricated sensor were better than those of non-electrochemical methods (Gupta et al., 2005; Amila Jeewantha et al., 2017; Kiani et al., 2018; Jin et al., 2021). When comparing with electrochemical methods, the HPLC/MS and HPLC are expensive, sophisticated and multi-process techniques, with the need for sample preparation, pre-filtration and extraction as well as temperature monitoring. In addition, the performance of our proposed DNA biosensor for sensing Vincristine displayed a comparable linear range and better detection limit and sensitivity when comparing with the other electrochemical methods. The detection limit of only Ref (Saify Nabiabad and Amini, 2020). developed for detection of Vincristine was superior to our sensor. The strength of our work than Ref (Saify Nabiabad and Amini, 2020). was the use of non-destructive, non-toxic and cost-effective modifiers (ds-DNA/PP/P-L CuO:Tb^3+^ NS) when comparing with Au nanoparticles, carbon nanotubes, monoclonal antibody. We also achieved lower limit of detection and broader linear range than others (Table 1). Accordingly, as-fabricated biosensor is potentially able to determine the trace amount of Vincristine in various media. Moreover, the electrode used for the sensor fabrication is a SPE that has various advantages like cost-effectiveness, facile modification, admirable accessibility and lower background current when comparing with other electrodes. As seen in Tables 1, the electrode as-fabricated for electrochemically bio-sensing Vincristine generally showed admirable properties for measurement speed, sensitivity, detection limit, linear range and sensitivity when compared to other methods reported in literature.

**TABLE 1:** Comparison of major characteristics of various methods for the determination of Vincristine.

Method	Dynamic ranges	Detection limits	Ref
High-performance liquid chromatography	.25–25.0 μg/ml	8.0 μg/ml	Gupta et al. (2005)
Spectrophotometric	5.0–50.0 μg/ml	2.108 μg/ml	Amila Jeewantha et al. (2017)
Liquid chromatography-tandem mass spectrometry	2.5–250.0 ng/ml	2.5 ng/ml	Jin et al. (2021)
High-performance liquid chromatography-UV detection	.05–5.0 mg/L	.15 mg/L	Kiani et al. (2018)
Voltammetry	.01–.2 µM	7.0 nM	Yong et al. (2004)
Voltammetry	.05–5.0 µM	.26 µM	Zhang et al. (2016b)
Voltammetry	.2–50.0 nM	.08 nM	Saify Nabiabad and Amini, (2020)
Amperometry	2.0–.1 µM	1.1 nM	Babkina and Ulakhovich, (2011)
Voltammetry	.01–400.0 µM	.21 nM and 2.39 nM	This work

### 3.7 Reproducibility, stability and interference analysis

**FIGURE 8:**
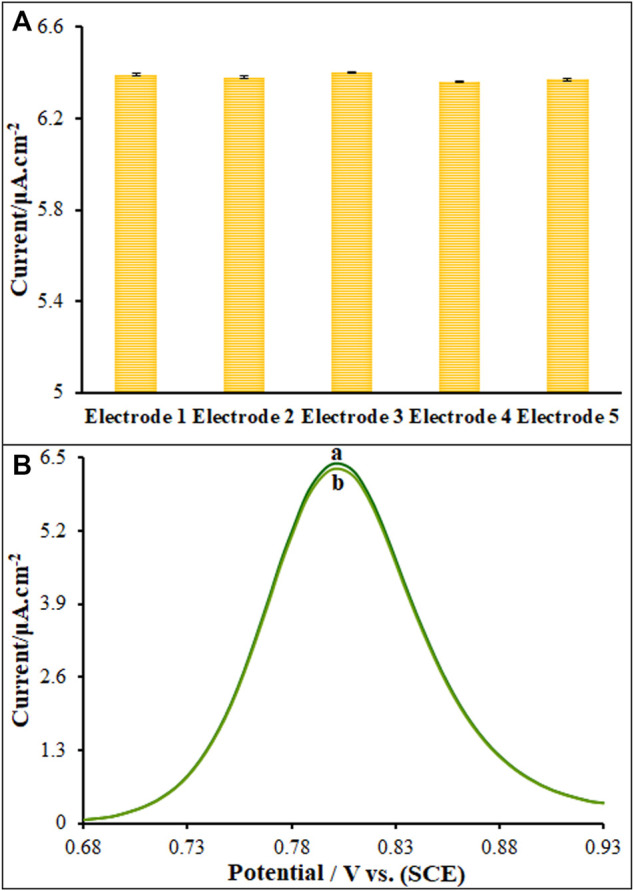
Current responses of Vincristine (50.0 μM) Five modified electrode fabricated **(B)** DPVs of modified electrode **(A)** (containing 50.0 μM of Vincristine) and **(B)** after 5 Weeks.

The peak currents of three study Vincristine was measured to determine the ds-DNA/PP/P-L CuO:Tb^3+^ NS/SPE reproducibility using five different sensors fabricated in the identical conditions (*n* = 5), the results of which showed the relative standard deviation (RSD) of 2.1% that means successful reproducibility of sensor (Figure 8A). The sensor was stored at 4°C for five consecutive weeks to determine the ds-DNA/PP/P-L CuO:Tb^3+^ NS/SPE stability, the results of which showed the maintenance of the peak current of 98.7% of its primary value that means successful stability of sensor (Figure 8B). The modified electrode was stored in ABS (pH 4.8) for Vincristine to test its stability for 10 days, followed by recording the DPVs and then comparing to pre-immersion DPVs. Based on the findings, the peak current was slightly reduced 99.2%, highlighting impressive stability of the sensor.

The interference determinations were carried out under the optimized conditions, consisting of metal ions and organic compounds. The tolerance limit for interferants was the highest concentration presenting a relative error of ˂±5.0% at the Vincristine concentration of 5.0 μM. The alanine, glycine, valine, dopamin, uric acid, ascorbic acid were the most abundant compounds in serum specimens along with Vincristine. According to the results these analytes separately (800-fold, 4.0 mM) could not interfere with the determination of Vincristine. In addition, the Ip values of Vincristine were not affected by 1000-fold concentrations (5.0 mM) of K^+^, Na^+^, Li^+^, F^−^, Ca^2+^ (Table 2). Accordingly, it can be claimed that none of the tested common interferants had a significant influence on the detection of Vincristine in the real study specimens using the as-fabricated ds-DNA/PP/P-L CuO:Tb^3+^ NS/SPE sensor.

**TABLE 2:** The selectivity test of ds-DNA/PP/P-L CuO:Tb^3+^ NS/SPE for the determination of Vincristine.

Species	Species tolerant limits (Winterference/W vincristine)
K^+^, Na^+^, Li^+^, F^−^, Ca^2+^	1,000
Alanine, Glycine, valine, Dopamin, Uric acid, Ascorbic acid	800

### 3.8 Real sample analysis

The practical potential of ds-DNA/PP/P-L CuO:Tb^3+^ NS/SPE was evaluated by sensing Vincristine in injection, human blood serum and urine samples using standard addition protocol. According to data (Table 3), the recovery rates were as satisfactory as 99.0%–103.3% that means the successful applicability of our Vincristine sensor. Moreover, the relative standard deviation (RSD) was lower than 4%, indicating a good precision of this method which can meet the requirement of nanoanalysis. Therefore, the developed electrochemical method is applicable to the determination of Vincristine.

**TABLE 3:** Determination of Vincristine in injection, serum and urine samples using ds-DNA/PP/P-L CuO:Tb^3+^ NS/SPE (*n* = 5).

Sample	Detected (µM)	Added (µM)	Found (µM)^a^	Recovery (%)
Vincristine injection	3.6	5.0	8.8 ± 2.1	102.3
		10.0	13.7 ± 2.7	100.7
Human Blood Serum	ND^b^	10.0	9.9 ± 3.6	99.0
		15.0	15.5 ± 1.9	103.3
Urine	ND^b^	20.0	20.1 ± 2.3	100.5
		25.0	24.9 ± 2.9	99.6

^a^ Mean ± standard deviation for *n* = 5.

^b^ Not detected.

### 3.9 Intercalation docking

**FIGURE 9:**
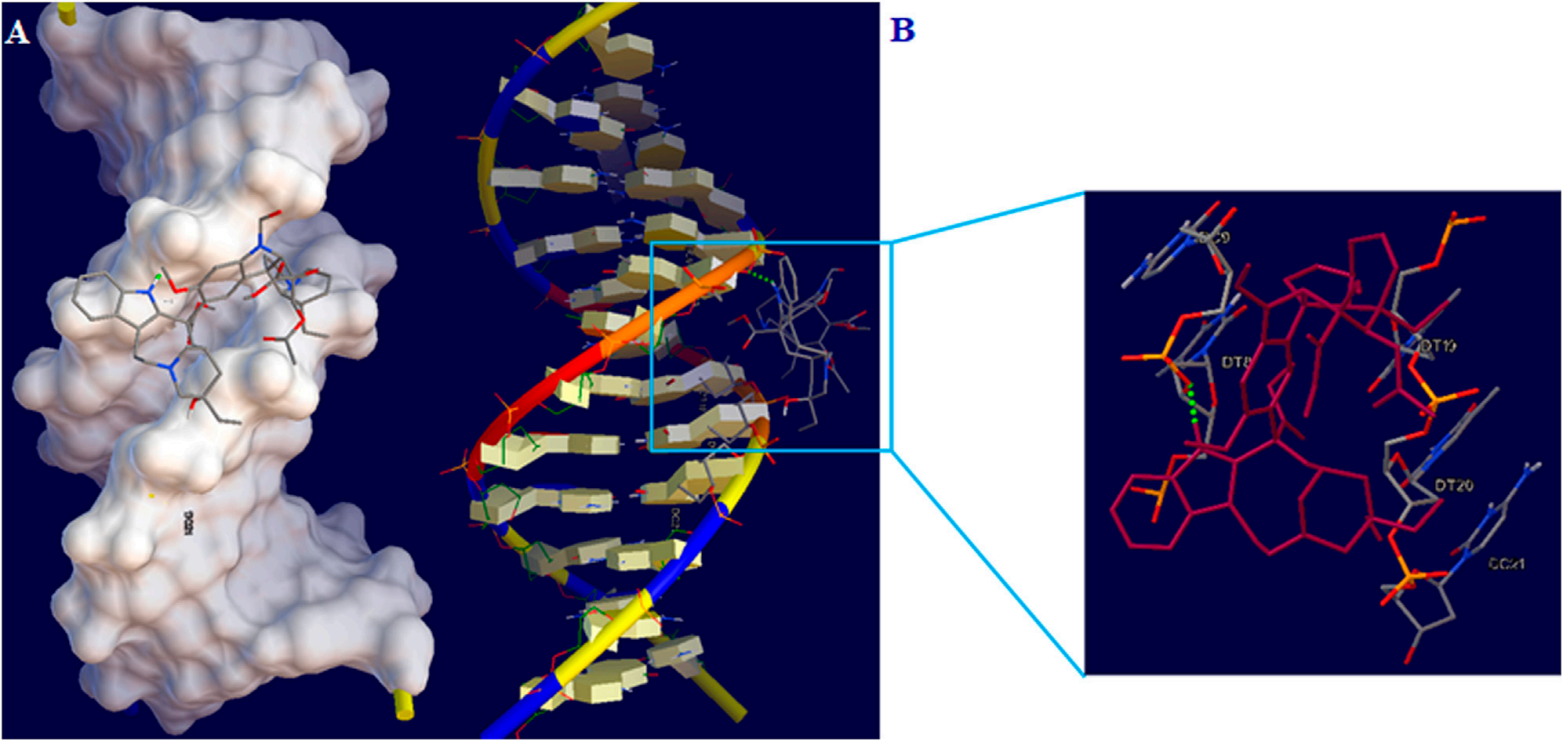
**(A)** Vincristine-DNA minor groove interaction **(B)** Geometrical disposition of vincristine in DNA minor groove.

Docking study was done to investigate the ideal interaction site and best compounds conformation on the DNA with the lowest energy. The lowest binding energy and Ki for the interaction of DNA with vincristine were obtained to be −6.08 kcal/mol and 35.18 μM, respectively. Studies indicated stabilization of vincristine at the DNA minor groove across one hydrogen bond with the nucleotides and hydrophobic interactions (Figure 9). Hydrogen (H) one bonds to nitrogen 48 of vincristine interacted with O3’ from thymine 8 (DT8). Moreover, the major contribution of hydrogen bond has been demonstrated in interacting between vincristine and DNA. According to the results of docking, vincristine can interact effectively with bases in the minor groove of DNA.

## 4 Conclusion

The P-L CuO:Tb^3+^ NS was prepared by the simple hydrothermal method. The physical characterization and structural properties are confirmed by the XRD, SEM and EDS analysis and discussed well. In addition, new DNA biosensor was designed using ds-DNA immobilization on the surface of PP/P-L CuO:Tb^3+^ NS/SPE. The DPV method was used to detect dsDNA-Vincristine interaction on the modified ds-DNA/PP/P-L CuO:Tb^3+^ NS/SPE surface. The factors affecting the performance of the biosensor such as DNA concentration (90 mg/ml), type of buffer solution (acetate buffer), and ds-DNA/Vincristine interaction time (12 min)) were optimized. In the analytical investigation of Vincristine using DPV, the modified electrode exhibited two linear responses in concentration ranges of .001–100.0 µM and 100.0–400.0 µM with detection limits of .021 and 2.39 nM, respectively. The experimental results and docking indicated that the binding mode of Vincristine and ds-DNA was minor groove. The practical potential of ds-DNA/PP/P-L CuO:Tb^3+^ NS/SPE was evaluated by sensing Vincristine in Vincristine injection, human blood serum and urine samples.

## Data Availability

The original contributions presented in the study are included in the article/supplementary material, further inquiries can be directed to the corresponding author.

## References

[B1] Amila Jeewantha H. Aleksei Ivanovich S. Pavel Mihailovich K. (2017). Validated spectrophotometric method for the estimation of vincristine and vinblastine. Int. J. Pharm. Pharm. Sci. 9, 78–86. 10.22159/ijpps.2017v9i4.16577

[B2] Anqi E. A. Li C. Dhahad H. A. Sharma K. Attia E. A. Abdelrahman A. (2022). Effect of combined air cooling and nano enhanced phase change materials on thermal management of lithium-ion batteries. J. Energy Storage 52, 104906. 10.1016/j.est.2022.104906

[B3] Babkina S. S. Ulakhovich N. A. (2011). Determination of pharmaceuticals based on indole alkaloids with amperometric DNA-sensors and enzyme immunoassay test-system. Anal. Lett. 44, 837–849. 10.1080/00032711003789975

[B4] Cao C. Wang J. Kwok D. Zhang Z. Cui F. Zhao D. (2022). webTWAS: a resource for disease candidate susceptibility genes identified by transcriptome-wide association study. Nucleic Acids Res. 50, D1123–D1130. 10.1093/nar/gkab957 34669946 PMC8728162

[B5] Chen C. Wang C. Zhao P. Zhang J. Ma D. Fei J. (2022). Determination of dopamine based on a temperature-sensitive PMEO2MA and Au@rGO-MWCNT nanocomposite-modified electrode. Analyst 147, 303–311. 10.1039/d1an02134f 34913448

[B6] Childress M. Q. Christian J. A. Ramos-Vara J. A. Rosen N. K. Ruple A. (2022). Greater baseline serum C-reactive protein concentrations are associated with reduced survival in dogs receiving cyclophosphamide, doxorubicin, vincristine, and prednisone chemotherapy for primary nodal diffuse large B-cell lymphoma. Vet. J. 289, 105911. 10.1016/j.tvjl.2022.105911 36202308

[B7] Dong J. Ren L. Zhang Y. Cui X. Hu P. Xu J. (2015). Direct electrodeposition of cable-like CuO@Cu nanowires array for non-enzymatic sensing. Talanta 132, 719–726. 10.1016/j.talanta.2014.10.027 25476370

[B8] Duan Z. Li C. Zhang Y. Yang M. Gao T. Liu X. (2023). Mechanical behavior and semiempirical force model of aerospace aluminum alloy milling using nano biological lubricant. Front. Mech. Eng. 18, 4. 10.1007/s11465-022-0720-4

[B9] Dung N. Q. Patil D. Jung H. Kim D. (2013). A high-performance nonenzymatic glucose sensor made of CuO–SWCNT nanocomposites. Biosens. Bioelectron. 42, 280–286. 10.1016/j.bios.2012.10.044 23208099

[B10] Ejaz A. Babar H. Ali H. M. Jamil F. Janjua M. M. Fattah I. R. (2021). Concentrated photovoltaics as light harvesters: Outlook, recent progress, and challenges. Sustain. Energy Technol. Assess. 46, 101199. 10.1016/j.seta.2021.101199

[B11] Fang Q. Liu X. Zeng K. Zhang X. Zhou M. Du J. (2022). Centrifuge modelling of tunnelling below existing twin tunnels with different types of support. Undergr. space 7, 1125–1138. 10.1016/j.undsp.2022.02.007

[B12] Farvardin N. Jahani Sh. Kazemipour M. Foroughi M. M. (2020). The synthesis and characterization of 3D mesoporous CeO_2_ hollow spheres as a modifier for the simultaneous determination of amlodipine, hydrochlorothiazide and valsartan. Anal. Methods 12, 1767–1778. 10.1039/d0ay00022a

[B13] Fathi Z. Jahani Sh. Shahidi Zandi M. Foroughi M. M. (2020). Synthesis of bifunctional cabbage flower–like Ho^3+^/NiO nanostructures as a modifier for simultaneous determination of methotrexate and carbamazepine. Anal. Bioanal. Chem. 412, 1011–1024. 10.1007/s00216-019-02326-8 31897563

[B14] Filippi-Chiela E. C. Eduardo Vargas J. Bueno e Silva M. M. Thome M. P. Lenz G. (2022). Vincristine promotes differential levels of apoptosis, mitotic catastrophe, and senescence depending on the genetic background of glioblastoma cells. Toxicol. Vitro 85, 105472. 10.1016/j.tiv.2022.105472 36116745

[B15] Foroughi M. M. Jahani Sh. Aramesh-Boroujeni Z. Vakili Fathabadi M. Hashemipour Rafsanjani H. Rostaminasab Dolatabad M. (2021). Template-free synthesis of ZnO/Fe_3_O_4_/Carbon magnetic nanocomposite: Nanotubes with hexagonal cross sections and their electrocatalytic property for simultaneous determination of oxymorphone and heroin. Michrochem. J. 170, 106679. 10.1016/j.microc.2021.106679

[B16] Foroughi M. M. Jahani S. (2021). Investigation of a high-sensitive electrochemical DNA biosensor for determination of Idarubicin and studies of DNA-binding properties. Microchim. J. 179, 107546. 10.1016/j.microc.2022.107546

[B17] Gao T. Li C. Wang Y. Liu X. An Q. Li H. N. (2022). Carbon fiber reinforced polymer in drilling: From damage mechanisms to suppression. Compos. Struct. 286, 115232. 10.1016/j.compstruct.2022.115232

[B18] Gupta M. M. Singh D. V. Tripathi A. K. Pandey R. Verma R. K. Singh S. (2005). Simultaneous determination of vincristine, vinblastine, catharanthine, and vindoline in leaves of catharanthus roseus by high-performance liquid chromatography. J. Chromatogr. Sci. 43, 450–453. 10.1093/chromsci/43.9.450 16212789

[B19] Hu Z. Liu H. (2015). Three-dimensional CuO microflowers as anode materials for Li-ion batteries. Ceram. Int. 41, 8257–8260. 10.1016/j.ceramint.2015.03.010

[B20] Iftikhar T. Asif M. Aziz A. Ashraf G. Jun S. Li G. (2021). Topical advances in nanomaterials based electrochemical sensors for resorcinol detection. Trends Environ. Anal. Chem. 31, e00138. 10.1016/j.teac.2021.e00138

[B21] Iftikhar T. Aziz A. Ashraf G. Xu Y. Li G. Zhang T. (2022). Engineering MOFs derived metal oxide nanohybrids: Towards electrochemical sensing of catechol in tea samples. Food Chem. 395, 133642. 10.1016/j.foodchem.2022.133642 35820273

[B22] Iftikhar T. Xu Y. Aziz A. Ashraf G. Li G. Asif M. (2021). Tuning electrocatalytic aptitude by incorporating α-MnO2 nanorods in Cu-MOF/rGO/CuO hybrids: Electrochemical sensing of resorcinol for practical applications. ACS Appl. Mat. Interfaces 13, 31462–31473. 10.1021/acsami.1c07067 34196524

[B23] Jahani Sh. Sedighi A. Toolabi A. Foroughi M. M. (2022). Development and characterization of La_2_O_3_ nanoparticles@snowflake-like Cu_2_S nanostructure composite modified electrode and application for simultaneous detection of catechol, hydroquinone and resorcinol as an electrochemical sensor. Electrochim. Acta 416, 140261. 10.1016/j.electacta.2022.140261

[B24] Jansi Rani B. Ravi G. Yuvakkumar R. Hasan Zinab M. Ravichandran S. Hong S. I. (2020). Binder free, robust and scalable CuO@GCE modified electrodes for efficient electrochemical water oxidation. Mat. Chem. Phys. 239, 122321. 10.1016/j.matchemphys.2019.122321

[B25] Jia D. Zhang Y. Li C. Yang M. Gao T. Said Z. (2022). Lubrication-enhanced mechanisms of titanium alloy grinding using lecithin biolubricant. Tribol. Int. 169, 107461. 10.1016/j.triboint.2022.107461

[B26] Jin Y. Li Y. Uddin M. E. Sparreboom A. Hu S. (2021). Rapid quantification of vincristine in mouse plasma using ESI-LC-MS/MS: Application to pharmacokinetic studies. J. Chromatogr. B 1168, 122591. 10.1016/j.jchromb.2021.122591 PMC7987214 33684722

[B27] Khan J. Ali G. Khurshid A. Saeed A. Ahmad S. Ullah N. (2022). Mechanistic efficacy assessment of selected novel methanimine derivatives against vincristine induced neuropathy: *In-vivo*, *ex-vivo* and in-silico correlates. Int. Immunopharmacol. 112, 109246. 10.1016/j.intimp.2022.109246 36116153

[B28] Kiani M. Qomi M. Hashemian F. Rajabi M. (2018). Multivariate optimization of solvent bar microextraction combined with HPLC-UV for determination of trace amounts of vincristine in biological fluids. J. Chromatogr. B 1072, 397–404. 10.1016/j.jchromb.2017.10.054 29174461

[B29] Kumar R. Ranjan N. Kumar V. Kumar R. Singh Chohan J. Piyush A. Y. (2022). Characterization of friction stir-welded polylactic acid/aluminum composite primed through fused filament fabrication. J. Mat. Eng. Perform. 31, 2391–2409. 10.1007/s11665-021-06329-4

[B30] Li A. L. Crystal J. D. Lai Y. Y. Sajdyk T. J. Renbarger J. L. Hohmann A. G. (2021). An adolescent rat model of vincristine-induced peripheral neuropathy. Neurobiol. Pain 10, 100077. 10.1016/j.ynpai.2021.100077 34841128 PMC8605395

[B31] Li B. Li C. Zhang Y. Wang Y. Jia D. Yang M. (2016). Grinding temperature and energy ratio coefficient in MQL grinding of high-temperature nickel-base alloy by using different vegetable oils as base oil. Chin. J. Aeronaut. 29, 1084–1095. 10.1016/j.cja.2015.10.012

[B32] Moarefdoust M. M. Jahani Sh. Moradalizadeh M. Motaghi M. M. Foroughi M. M. (2022). A DNA biosensor based on a raspberry-like hierarchical nano-structure for the determination of the anticancer drug nilotinib. ChemistrySelect 11, e202100261. 10.1002/open.202100261 35333006 PMC8950773

[B33] Moarefdoust M. M. Jahani Sh. Moradalizadeh M. Motaghi M. M. Foroughi M. M. (2022). A DNA biosensor based on a raspberry-like hierarchical nano‐structure for the determination of the anticancer drug nilotinib. ChemistryOpen 11, e202100261. 10.1002/open.202100261 35333006 PMC8950773

[B34] Mujib A. Fatima S. Malik M. Q. (2022). Cryo-derived plants through embryogenesis showed same levels of vinblastine and vincristine (anticancer) in Catharanthus roseus and had normal genome size. Sci. Rep. 12, 16635. 10.1038/s41598-022-20993-z 36198853 PMC9534890

[B35] Núñez C. Triviño J. J. Arancibia V. (2021). A electrochemical biosensor for As(III) detection based on the catalytic activity of Alcaligenes faecalis immobilized on a gold nanoparticle–modified screen–printed carbon electrode. Talanta 223, 121702. 10.1016/j.talanta.2020.121702 33298256

[B36] Park H. J. Choi N.-J. Kang H. Jung M. Y. Park J. W. Park K. H. (2014). A ppb-level formaldehyde gas sensor based on CuO nanocubes prepared using a polyol process. Sensors Actuators B Chem. 203, 282–288. 10.1016/j.snb.2014.06.118

[B37] Saify Nabiabad H. Amini M. (2020). Fabrication of an impedimetric immunosensor for screening and determination of vincristine in biological samples. J. Anal. Chem. 75, 1094–1101. 10.1134/s1061934820080092

[B38] Tian Y. Yu L. Wang W. P. Zhang X. Peng W. (2015). CuO nanoparticles on sulfur-doped graphene for nonenzymatic glucose sensing. Electrochim. Acta 156, 244–251. 10.1016/j.electacta.2015.01.016

[B39] Uma Maheswari R. Jansi Rani B. Ravi G. Yuvakkumar R. Ameen F. Al-Sabri A. (2018). Structural, morphological, optical and antibacterial properties of pentagon CuO nanoplatelets. J. Sol-Gel Sci. Tech. 87, 515–527. 10.1007/s10971-018-4773-0

[B40] Vakili Fathabadi M. Hashemipour Rafsanjani H. Foroughi M. M. Jahani Sh. Arefi Nia N. (2020). Synthesis of magnetic ordered mesoporous carbons (OMC) as an electrochemical platform for ultrasensitive and simultaneous detection of thebaine and papaverine. J. Electrochem. Soc. 167, 027509. 10.1149/1945-7111/ab6446

[B41] Yang C. Wang J. Xiao F. Xintai S. (2014). Microwave hydrothermal disassembly for evolution from CuO dendrites to nanosheets and their applications in catalysis and photo-catalysis. Powder Technol. 264, 36–42. 10.1016/j.powtec.2014.05.012

[B42] Yang M. Li C. Zhang Y. Wang Y. Li B. Jia D. (2017). Research on microscale skull grinding temperature field under different cooling conditions. Appl. Therm. Eng. 126, 525–537. 10.1016/j.applthermaleng.2017.07.183

[B43] Yang Z. Feng J. Qiao J. Yan Y. Yu Q. Sun K. (2012). Copper oxide nanoleaves decorated multi-walled carbon nanotube as platform for glucose sensing. Anal. Methods 4, 1924. 10.1039/c2ay25283j

[B44] Yong Y. Jing-Bo H. Jun S. Qi-Long L. (2004). Study on the electrochemical behaviors of vincristine and the interaction of vincristine with tubulin. Acta Chim. Sin. 62, 137–141.

[B45] Zhang H. Zou Q. Ju Y. Song C. Chen D. (2022). Distance-based support vector machine to predict DNA N6-methyladenine modification. Curr. Bioinform. 17, 473–482. 10.2174/1574893617666220404145517

[B46] Zhang Y. B. Li C. H. Yang M. Jia D. Z. Wang Y. Li B. (2016). Experimental evaluation of cooling performance by friction coefficient and specific friction energy in nanofluid minimum quantity lubrication grinding with different types of vegetable oil. J. Clean. Prod. 139, 685–705. 10.1016/j.jclepro.2016.08.073

[B47] Zhang Y. Zheng J. Guo M. (2016). Preparation of molecularly imprinted electrochemical sensor for detection of vincristine based on reduced graphene oxide/gold nanoparticle composite film. Chin. J. Chem. 34, 1268–1276. 10.1002/cjoc.201600582

[B48] Zheng B. Liu G. Yao A. Xiao Y. Juan D. Guo Y. (2014). A sensitive AgNPs/CuO nanofibers non-enzymatic glucose sensor based on electrospinning technology. Sensors Actuators B 195, 431–438. 10.1016/j.snb.2014.01.046

[B49] Zhou Q. Umar A. Sodki E. M. Amine A. Xu L. Gui Y. (2018). Fabrication and characterization of highly sensitive and selective sensors based on porous NiO nanodisks. Sens. Actuators B 259, 604–615. 10.1016/j.snb.2017.12.050

